# Evaluating Multiprofessional Caseload Review in the Community Mental Health Team One Year On: Improving Patient Flow and Creating a More Responsive Service

**DOI:** 10.1192/bjo.2024.459

**Published:** 2024-08-01

**Authors:** Zarina Anwar, Olivia McClure

**Affiliations:** Leicestershire Partnership NHS Trust, Leicester, United Kingdom

## Abstract

**Aims:**

To evaluate the effectiveness and sustainability of multidisciplinary outpatient caseload reviews in the community mental health team (CMHT).

**Methods:**

Caseload review for all patients under the outpatient clinics within South Leicestershire CMHT commenced in August 2022. A consultant psychiatrist and senior nurse spent 2–4 hours weekly reviewing each patient's electronic record chronologically from those waiting the longest for an appointment guided by a template including variables such as stability, risk and medication. Based on clinical need, the patient may be offered an outpatient appointment for ongoing treatment or review for discharge, nurse discharge clinic or transfer to another service.

This process is now embedded into routine clinical work, and momentum sustained by clinical and operational leadership roles within the team. The cycle is iterative and ongoing to ensure patients new to the service are included and flow from referral to discharge maintained.

**Results:**

Between August 2022 to January 2024, 1460 out of a total of 1699 caseload reviews were completed. 622 (42%) of these are identified as suitable to be reviewed for discharge.

Of those, 256 (41%) were suitable for nurse discharge clinic, and 366 (59%) by a medic.

110 patients received an outpatient appointment following nurse discharge clinic, clinically indicated in 25.

Average additional wait time for an outpatient appointment has reduced from 34 weeks (September 2022) to 22 weeks (January 2024).

**Conclusion:**

Consultant Psychiatrists in the CMHT frequently hold high outpatient caseloads with associated delays to care and treatment, and limited capacity and flexibility to respond dynamically to patient need contributing to reduced job satisfaction and burnout. Embedding multiprofessional caseload review into routine work creates greater capacity and responsiveness, reducing outpatient wait times and improving quality of care by earlier identification of those needing more expeditious review. Continuing this in an iterative cycle aligns with key principles of community transformation in the NHS Long Term Plan ensuring effective caseload management and fostering a more dynamic and responsive approach to meet patient need. Engaging senior clinicians and administrative staff is critical to successful implementation and close joint working has a positive ripple effect on team cohesion, morale and shared clinical decision making. The benefits are recognised at Trust board level with funding secured from the local Integrated Care Board to implement caseload reviews across all CMHTs within Leicestershire Partnership NHS Trust.

